# Brief Intervention in Primary Care Settings: A Primary Treatment Method for At-Risk, Problem, and Dependent Drinkers

**Published:** 1999

**Authors:** Michael Fleming, Linda Baier Manwell

**Affiliations:** Michael Fleming, M.D., M.P.H., is a professor of family medicine at the University of Wisconsin–Madison and director of the University of Wisconsin Center for Addiction Research and Education, Madison, Wisconsin. Linda Baier Manwell is deputy director of the University of Wisconsin Center for Addiction Research and Education, Madison, Wisconsin

**Keywords:** primary health care, intervention, risk factors, problematic AOD (alcohol and other drug) use, AOD dependence, amount of AOD use, treatment outcome, AOD abstinence, drug therapy, psychological counseling, treatment barriers, physician, AOD education, health care delivery, health care cost, social cost of AODU (alcohol and other drug use), literature review

## Abstract

Primary health care providers identify and treat many patients who are at risk for or are already experiencing alcohol-related problems. Brief interventions—counseling delivered by primary care providers in the context of several standard office visits—can be a successful treatment approach for many of these patients. Numerous trials involving a variety of patient populations have indicated that brief interventions can reduce patients’ drinking levels, regardless of the patients’ ages and gender. In clinical practice, brief interventions can help reduce the drinking levels of nondependent drinkers who drink more than the recommended limits, facilitate therapy and abstinence in patients receiving pharmacotherapy, and enhance the effectiveness of assessment and treatment referral in patients who do not respond to brief interventions alone. Despite the evidence for their usefulness, however, brief interventions for alcohol-related problems have not yet been widely implemented in primary care settings.

Most Americans consume alcohol at least occasionally, and results from the National Household Survey (U.S. Department of Health and Human Services 1998) suggest that as many as 40 million Americans drink more than the moderate drinking levels recommended by the National Institute on Alcohol Abuse and Alcoholism (NIAAA) (1995). These drinkers are considered at-risk, problem, or dependent alcohol users (for definitions of different types of alcohol use, see textbox on p. 129).

**Defining Moderate, At-Risk, Problem, and Dependent Alcohol Use**

According to guidelines published by the National Institute on Alcohol Abuse and Alcoholism in 1995, *moderate*, or low-risk, alcohol use is defined as consumption of no more than two standard drinks per day for men and no more than one standard drink per day for women and people over age 65. A standard drink is defined as one 12-ounce beer or wine cooler, one 5-ounce glass of wine, or 1.5 ounces of distilled spirits. Each of these standard drinks contains approximately 0.5 ounce, or 14 grams, of pure alcohol.

*At-risk* alcohol use is defined as consumption of more than 7 drinks per week or more than 3 drinks per occasion for women and more than 14 drinks per week or more than 4 drinks per occasion for men. A positive response to one or more questions on the four-item CAGE questionnaire (see footnote 1 below) also may indicate at-risk use. *Problem* alcohol use is defined as one or more positive responses to the CAGE questionnaire and evidence of alcohol-related medical or behavioral problems. *Dependent* use is defined as either three or four positive responses to the CAGE questionnaire and/or evidence of one or more symptoms of alcohol dependence (i.e., compulsion to drink, impaired control over drinking, withdrawal symptoms, drinking to relieve withdrawal, and increased tolerance to alcohol).

Many people who are at risk for, or who are already experiencing, alcohol-related social and medical problems do not consult alcoholism treatment specialists but receive their health care from a primary care provider. Consequently, primary care settings offer an important opportunity to identify and treat people with potential drinking problems. Epidemiological analyses underscore the notion that primary care settings are pivotal in helping people with alcohol-related problems. For example, a prevalence study conducted in primary care settings found that 20 percent of male patients and 10 percent of female patients who came to see their physicians met the criteria for at-risk, problem, or dependent alcohol use (Manwell et al. 1998). Furthermore, 70 percent of American adults visit a physician at least once every 2 years for a routine physical examination or a specific health problem, suggesting that primary care providers potentially can identify and treat a substantial proportion of people experiencing alcohol-related adverse effects.

One treatment method that has proved to be effective in primary care settings is physician-delivered brief intervention (Fleming et al. 1997, 1999; Ockene et al. in press). This article describes the brief intervention approach, its essential components, and evidence for its effectiveness. In addition, the article discusses the current application of brief intervention in the U.S. health care system as well as barriers to its implementation. Finally, the article proposes measures that may help overcome those barriers.

## Essential Elements of Brief Intervention

The term “brief intervention” refers to a time-limited, patient-centered counseling strategy that focuses on changing patient behavior and increasing patient compliance with therapy. Although this article focuses on the use of brief intervention for changing alcohol use patterns, this approach is not unique to the treatment of alcohol problems. In fact, physicians and other health care professionals widely employ this technique to help patients change a variety of behaviors (e.g., to modify dietary habits; stop smoking; and reduce weight, cholesterol levels, or blood pressure).

In general, brief intervention consists of the following five essential steps (also see textbox on p. 130):

- *Assessment and direct feedback.* The health care provider assesses the patient’s alcohol use and the presence of alcohol-related problems using, for example, the four-item CAGE questionnaire.^1^ The provider then expresses his or her concern regarding the patient’s drinking pattern, linking, when appropriate, the alcohol use to a medical problem, such as high blood pressure (i.e., hypertension) or inflammation of the stomach lining (i.e., gastritis).
- *Negotiation and goal setting.* The treatment provider and patient agree on a mutually acceptable goal for reducing alcohol use (e.g., the moderate drinking levels recommended by the NIAAA [1995]).
- *Behavioral modification techniques.* The health care provider helps the patient to identify high-risk situations in which drinking will likely occur, such as family celebrations or stressful situations at work. The provider also familiarizes the patient with coping techniques for managing such high-risk situations and with ways for establishing a support network to help in this process.
- *Self-help-directed bibliotherapy.* For reinforcement, the health care provider supplies the patient with informational materials on alcohol use and its associated problems as well as on behavioral modification exercises.
- *Followup and reinforcement.* To ensure the long-term effectiveness of the brief intervention, the health care provider establishes a system for conducting supportive telephone consultation and followup visits with the patient.

The Five Essential Steps of Brief Intervention*

**Step I. Assessment and Direct Feedback**

Ask questions regarding alcohol consumption.

Ask CAGE questions.**

Assess medical, behavioral, and dependence problems.

- As your physician, I am concerned about how much you drink and how it is affecting your health.
- Less than 10 percent of men drink as much as you do.
- You are drinking alcohol at a level that puts you at serious risk for a number of alcohol-related problems.

**Step II. Negotiation and Goal Setting**

- You need to reduce your drinking.
- What do you think about cutting down to three drinks two to three times per week?
- Can you reduce your alcohol use for the next month?

**Step III. Behavioral Modification Techniques**

- Here is a list of situations when people drink and sometimes lose control of their drinking. Let’s talk about ways you can avoid these situations.
- Can you identify a family member or a friend who can help you?
- What are the things you like about drinking?
- What are some of the things you don’t like about your alcohol use?

**Step IV. Self-Help-Directed Bibliotherapy**

- I would like you to review this booklet and bring it with you to your next visit. It would be helpful if you would complete some of the exercises in the booklet.

**Step V. Followup and Reinforcement**

- I would like you to return to the clinic in 1 month to see if you have been able to change your drinking.
- My nurse will call you in 2 weeks to check on your progress.
- I would like you to keep track of your drinking by using these diary cards. Bring them with you to your followup visit in 1 month.

*The italicized items indicate sample statements or questions that a primary care physician might use with his or her patients.**See footnote 1 on p. 129.

A health care provider usually can conduct brief intervention incorporating these components during a standard 5- to 10-minute office visit. The number of visits required to ensure treatment success can vary, but studies suggest that three to four visits, or a combination of clinic visits and followup telephone consultations, can increase the effectiveness of the brief intervention (Wallace et al. 1988; Kristenson et al. 1983; Anderson and Scott 1992; Fleming et al. 1997). Intervention workbooks that guide the health care provider through brief intervention and “drinking diary cards” that help the patient track his or her alcohol consumption can be useful tools for focusing the provider-patient discussion and for facilitating behavior change (Fleming et al. 1997).^2^ Finally, more extensive counseling sessions with a clinic health educator or nurse can supplement the brief intervention delivered by the primary care provider.

## Evidence of Brief Intervention’s Effectiveness in Primary Care Settings

Clinical trials have demonstrated that brief intervention administered in a variety of treatment settings can reduce alcohol use for at least 12 months in patients who are not alcohol dependent. At least 20 trials conducted in medical clinics have been reported in the medical literature. Meta-analyses performed by Bien and colleagues (1993), Kahan and colleagues (1995), and Wilk and colleagues (1997) found that most brief intervention trials showed a positive outcome, as indicated by reduced consumption levels. Furthermore, these analyses suggested that clinicians could expect 10 to 30 percent of their patients to change their drinking behaviors as a result of brief intervention.

Few published trials of brief intervention have been performed exclusively in community-based primary care settings. However, studies conducted by Wallace and colleagues (1988), Israel and colleagues (1996), Fleming and colleagues (1997, 1999), and Ockene and colleagues (in press) have presented compelling evidence to support the effectiveness of brief intervention in primary care settings. Another trial of brief intervention, conducted in a variety of settings by the World Health Organization (WHO) Brief Intervention Study Group (1996), also found positive outcomes associated with brief intervention. The validity of these findings is further supported by the fact that all these trials included large sample sizes and highly diverse patient populations. For a summary of the designs and major findings of these trials, see the table on pp. 132–133.

Brief intervention appears to be effective for both men and women as well as across all age groups. To date, only one study has suggested that brief intervention may be more effective for women than for men (Sanchez-Craig 1990). Conversely, the six trials mentioned in the previous paragraph all found that brief intervention led to similar reductions in alcohol consumption for men and women. Furthermore, when Fleming and colleagues (1997) analyzed the effectiveness of brief intervention for patients of different ages in Project TrEAT (Trial for Early Alcohol Treatment), they found no difference in treatment effectiveness across age groups. However, only one trial has been conducted exclusively with older adults. In that study, called Project GOAL (Guiding Older Adult Lifestyles), brief intervention led to a 20-percent reduction in drinking levels in a sample of 158 older adults ages 65 to 85 (Fleming et al. 1999).

Brief intervention can reduce not only the drinking levels of problem drinkers but also their health care utilization for related medical conditions. For example, as part of a study conducted in the late 1970s that focused on the prevention of cardiovascular disease, all men ages 46 to 53 residing in Malmo, Sweden, were invited to participate in a screening for cardiovascular disease, diabetes, and heavy drinking (Kristenson et al. 1983). The study identified 585 men with elevated blood levels of the enzyme gamma-glutamyl transferase (GGT), an indicator of long-term excessive alcohol consumption. The men were randomly assigned to either an experimental or control group. Over a study period of 12 months, the men in the experimental group received a brief intervention consisting of a consultation with their physician every 3 months, a monthly GGT test, and monthly contact with a nurse. The control group only received a letter with their initial GGT results and advice to reduce their alcohol consumption. The study found long-term (i.e., for 5 years after study entry) reductions in hospital days, sick days, and mortality in the experimental group compared with the control group. Project TrEAT, which included 774 patients ages 18 to 65 who were randomly assigned either to a brief intervention group or to a control group, also reported a significant decrease in hospital days in the intervention group compared with the control group (Fleming et al. 1997). At the 1-year followup, the control group had required twice as many hospital days as had the intervention group. For a more detailed description of both trials, see the table.

In most trials on the effectiveness of brief interventions, physicians delivered the interventions. For example, in the study by Wallace and colleagues (1988), 47 physicians throughout Great Britain were trained to administer the brief intervention protocol in their practices. Similarly, Project TrEAT recruited 64 family physicians from 10 counties in southern Wisconsin to participate in training programs and successfully complete the required research protocol (Fleming et al. 1997). However, other health care professionals, such as nurse practitioners, also can be taught to successfully conduct brief intervention. For example, Project HEALTH, which was conducted in Massachusetts and included 46 physicians and nurse practitioners (26 attending physicians, 12 resident physicians, and 8 nurse practitioners), had similar success rates in teaching clinicians to conduct brief intervention, as did the trials involving only physicians (Ockene et al. in press).

### Unanswered Questions

Although many of the clinical trials conducted to date have strongly supported the notion that brief intervention can be an effective tool for reducing the drinking levels of people at risk for or experiencing alcohol-related problems, numerous questions remain, including the following:

- Does brisef intervention reduce overall health care costs?
- Does brief intervention reduce alcohol use for more than 12 months, the most frequent followup period in the trials?
- Does brief intervention delivered in emergency departments and hospitals, rather than by primary care physicians, reduce rates of alcohol-related problems, such as accidents and injuries?
- Does brief intervention for women at risk for alcohol use during pregnancy reduce the rates of fetal alcohol exposure?
- Does brief intervention work better when combined with pharmacotherapy?
- Is brief intervention more or less effective when performed by the patient’s personal health care team rather than by a researcher who has no prior relationship with the patient?
- How can physicians and managed care organizations be convinced to implement brief intervention in primary care settings?

Many of these questions are being addressed by a dozen trials funded by the NIAAA and other Federal agencies that are either ongoing or have been completed recently but have not yet been reported in the literature.

**Table:** Design and Major Results of Selected Brief Intervention Trials

Researchers and Study/Study Site	Selection Process	Population of Interest and Sample Size	Intervention Protocol and Drop-Out Rates	Results
Kristenson et al. 1983 Malmo, Sweden (community health centers)	Men participating in a screening for cardiovascular disease, diabetes, and heavy drinking	Men ages 46–53 Exp = 317 Cont = 268	Exp: physician consultation every 3 mo, monthly GGT test, monthly nurse contact Cont: informed of GGT by letter, told to cut down, had further liver tests after 2 yr Followup: 2, 4, and 5 yr. Drop out: unknown	GGT values reduced in both groups; significant reduction in sick days, hospital days, and mortality in exp compared with cont; alcohol use not determined
Chick et al. 1985 Royal Edinburgh Hospital, Edinburgh, Scotland	Consecutive admissions of at least 48-h duration	Men ages 18–65 in one of four medical wards Exp = 78 Cont = 78	Exp: counseling with nurse up to 1 h, self-help booklet Cont: nurse assessment Followup: 12 mo Drop out: exp = 12%, cont = 18%	No significant difference in alcohol consumption at 12 mo; reduced alcohol-related problems and reduced GGT in exp
Wallace et al. 1988 MRC Trial, England (rural and small urban general practices)	Mailed and in-practice questionnaires Consumption: men—35+ units/wk, women—21+ units/wk	Male and female patients in general medical practices Exp: 319 men, 131 women Cont: 322 men, 137 women	Exp: physician assessment, booklet, told to cut down Cont: no advice unless requested by patient or evidence of liver impairment Followup: 6 and 12 mo Drop out after 6 mo: men—15%, women—13% Drop out after 12 mo: men—19%, women—17%	At 6 and 12 mo, significant reduction in drinking for exp compared with cont; GGT and blood pressure reduced in exp men
Persson and Magnusson 1989 Sweden (outpatient clinics)	Questionnaires and GGT levels Consumption: men—200+ g/wk, women—150+ g/wk, GGT greater than 0.6	Patients ages 15–70 attending outpatient clinics Exp = 36 Cont = 42	Exp: physician interview, monthly nurse followup, quarterly physician followup, told to cut down Cont: initial questionnaire, no discussion on consumption, blood samples at 12 mo Followup: 12 mo Drop out: 0%	Consumption, triglycerides, GGT, and sick days decreased in exp; sick days increased in cont; no followup alcohol data available for cont
Nilsson 1991 Tromso, Norway	Questionnaire and GGT levels	Male and female patients ages 20–62 Exp 1 = 113 Exp 2 = 113 Cont = 112	Exp 1: brief health counseling Exp 2: counseling sessions specifically alcohol focused Followup: 12 mo Drop out: 5%	Significant differences between exp and cont for alcohol use and GGT levels; no differnces between exps
Anderson and Scott 1992 Oxford Regional Health Authority, England (eight group practices)	Self-administered questionnaires disseminated in office and by mail Consumption: 350–1,050	Men ages 17–69 in general medical practice settings Exp = 80 Cont = 74	Exp: physician advice for 10 min, self-help booklet Cont: no advice, self-help booklet Followup: 12 mo Drop out: exp—24%, cont—36%	Exp showed significant decrease in consumption compared with cont
Maheswaran et al. 1992 Hypertensive Clinic, Dudley Road Hospital, England	Referral by general practitioners Consumption: 20+ units/wk	Men drinking more than 20 units/wk Exp = 22 Cont = 23	Exp: 10- to 15-min sessions advising to cut down or abstain, advice reinforced at subsequent visits Cont: no intervention Followup: 8 wk Drop out: exp—5%, cont—13%	Significantly greater reduction in alcohol consumption and in standing diastolic blood pressure in exp
Marlatt et al. 1998 University of Washington, Seattle, WA (student health services)	Students screened during senior year of high school and then randomly assigned during freshman year of college	Students ages 18–25 160 men 188 women	Exp: health educator, 4-session intervention Cont: routine medical care Followup: 24 mo Drop out: 13%	Significant reduction in both drinking rate and harmful consequences
Israel et al. 1996 Cambridge, Ontario (primary care practices)	TRAUMA scale instrument given to patients ages 30–60	Males and females attending family medicine clinics Exp = 52 Cont = 53	Exp: 20-min counseling with nurse educator every 2 mo for 1 yr, self-help pamphlet Cont: brief advice, self-help pamphlet Followup: 12 mo Drop out: 30%	Both groups showed reduction in alcohol consumption; exp showed significant reduction in psychosocial problems, physician visits, and GGT
WHO Brief Intervention Study Group 1996 WHO 10-nation study various settings	Interviews at ERs, hospitals, clinics, workplaces, and health-screening agency Consumption: men—350+ g/wk, women—225+ g/wk	Cross-cultural 1,260 men 299 women	Exp 1: 20-min interview, 5 min of advice, pamphlet Exp 2: interview, 5 min of advice, 15 min of counseling, pamphlet Control: interview Followup: minimum 6 mo, average 9 mo Drop out: 25%, varying by center	Significant reduction in alcohol use and binge drinking in exps for males: significant reductions for all groups for women; exps 1 and 2 were equally effective
Fleming et al. 1997 Project TrEAT Southern Wisconsin (64 community-based primary care physicians in 10 counties)	In-office questionnaires given to all patients ages 18–65 with regular appointments Consumption: men—15+ drinks/wk, women—12+ drinks/wk, binge drinking, positive CAGE responses	Men and women ages 18–65 attending primary care clinics Exp = 392 Cont = 382	Exp: two 15-min physician visits, self-help book, drinking diary cards, drinking contract, two nurse-followup calls Cont: general health booklet Followup: 6, 12, 24, 36, and 48 mo Drop out: 7% at 12 mo, 11% at 24 mo, 17% at 48 mo	Significant reduction in 7-day alcohol use, binge drinking, frequency of excessive drinking, and length of hospitalization in exp compared with cont
Fleming et al. 1999 Project GOAL Southern Wisconsin (43 community-based primary care physicians in 10 counties)	In-office questionnaires given to all patients age 65 or older with regular appointments Consumption: men—12+ drinks/wk, women—9+ drinks/wk, binge drinking, positive CAGE responses	Men and women ages 65 or older attending primary care clinics Exp = 87 Cont = 71	Exp: two 15-min physician visits, self-help book, drinking diary cards, drinking contract, two nurse-followup calls Cont: general health booklet Followup: 3, 6, 12, and 24 mo Drop out: 8% at 12 mo, 12% at 24 mo	Significant reduction in 7-day alcohol use, episodes of binge drinking, and frequency of excessive drinking in exp compared with cont
Ockene et al. in press Project Health University of Massachusetts Memorial Healthcare Inc. (four primary care internal medicine practice sites)	Adults with regular appointments interviewed by phone, mail, or during visit to primary care center Consumption: men—13+ drinks/wk, women—10+ drinks/wk, binge drinking, positive CAGE responses	Men and women ages 21–70 attending internal medicine clinics Exp = 274 Cont = 256	Exp: two 5- to 10-min physician or nurse practitioner visits, general health booklet Cont: general health booklet Followup: 6 mo Drop out: 9%	Significant reduction in weekly alcohol consumption and excessive drinking in exp compared with cont

Cont = control group not receiving brief intervention; exp = experimental group receiving specified brief intervention; g/wk = grams of alcohol per week; GGT = gamma-glutamyl transferase, an enzyme that serves as an indicator of excessive long-term alcohol consumption; h = hour(s); min = minute(s); mo = month(s); yr = year(s).

Additional issues have been raised by several brief intervention trials that found only minimal differences between experimental groups receiving brief intervention and control groups (Richmond et al. 1995; Senft et al. 1997; Burge et al. 1997; Chang et al. in press). Each of those trials found large reductions in alcohol use in both the experimental and control groups. Several factors may explain this observation. First, the research procedure itself, which included questions about alcohol use on multiple occasions for all study subjects, may have exerted an intervention effect. In that case, simply drawing attention to a patient’s excessive drinking may have positively influenced the patient’s drinking behavior. Second, the reduction in alcohol use could result from a phenomenon called “regression to the mean,” which occurs because of the natural variability in alcohol use. This means that some patients will have been screened and enrolled in the study at time points at which their alcohol consumption was abnormally high compared with their average consumption over time. In such patients, subsequent consumption measurements will tend to gravitate back to their average consumption levels. Third, reduced drinking levels in the control subjects may be related to normal changes in alcohol use that occur in any person over time.

## The Role of Brief Intervention in the Treatment of People With Alcohol Problems

**Figure 1:**
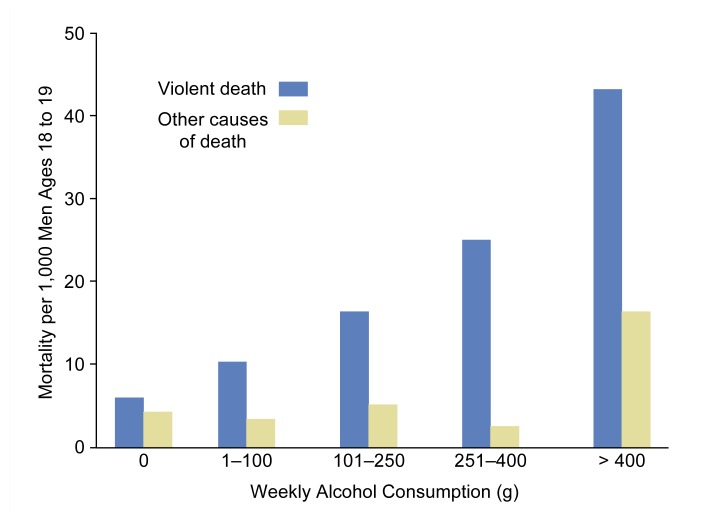
The relationship between alcohol use (grams [g] of alcohol per week) and mortality (deaths per 1,000), both from violence (blue bars) and from causes other than violence (yellow bars), in young men ages 18 to 19. The risk of violent death increases steadily with increasing alcohol consumption. Conversely, the risk of death from other causes remains relatively low at a consumption level less than 400 g alcohol (or 28 standard drinks) per week but increases substantially with a weekly alcohol consumption of more than 400 g. Source: Andreasson et al. 1988.

**Figure 2:**
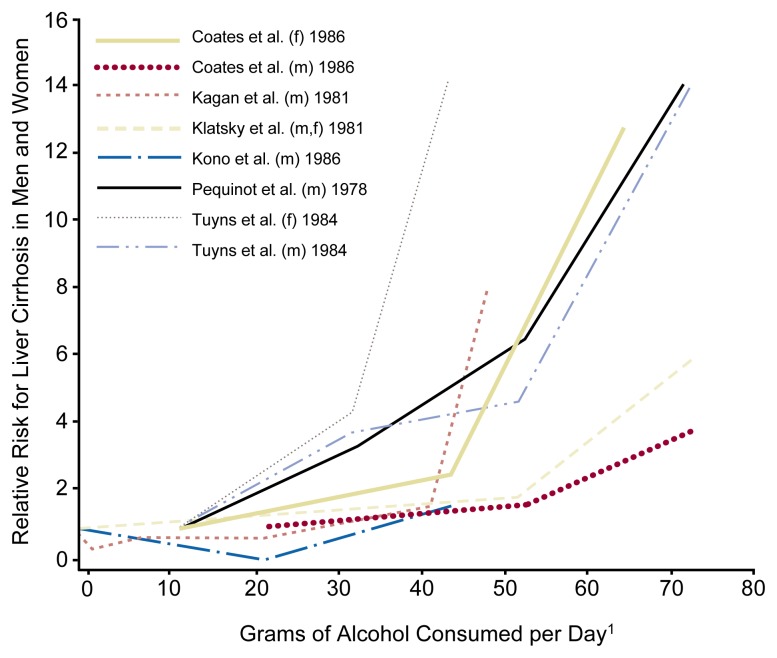
The relationship in men and women between alcohol use (i.e., grams of alcohol per day [g/day]) and the relative risk of developing liver cirrhosis. The lines represent the results of six different studies. In each of these studies, the risk for liver cirrhosis increased with increasing alcohol consumption. ^1^Data for alcohol consumption greater than 70 g/day are not shown. f = female subjects; m = male subjects. NOTE: References for the six studies are as follows: Coates, R.A.; Halliday, M.L.; Rankin, J.G.; Feinman, S.V.; and Fisher, M.M. Risk of fatty infiltration or cirrhosis of the liver in relation to ethanol consumption: A case-control study. *Clinical and Investigative Medicine—Medecine Clinique et Experimentale* 9:26–32, 1986. Kagan, A.; Yano, K.; Roads, G.; and McGee, D.L. Alcohol and cardiovascular disease: The Hawaiian experience. *Circulation* 64(3):III27–31, 1981. Klatsky, A.L.; Friedman, G.D.; and Seigelaub, A.B. Alcohol and mortality: A ten-year Kaiser-Permanente experience. *Annals of Internal Medicine* 95:139–145, 1981. Kono, S.; Ikeda, M.; Tokudome, A.; Nishizumi, M.; and Kuratsune, M. Alcohol and mortality: A cohort study of male Japanese physicians. *International Journal of Epidemiology* 15:527–532, 1986. Pequinot, G.; Tuyns, A.J.; and Berta, J.L. Ascitic cirrhosis in relation to alcohol consumption. *International Journal of Epidemiology* 7:113–120, 1978. Tuyns, A.J., and Pequinot, G. Greater risk of ascitic cirrhosis in females in relation to alcohol consumption. *International Journal of Epidemiology* 14:53–57, 1984. SOURCE: Anderson et al. 1993.

Brief intervention can be useful for the treatment of at-risk, problem, and dependent drinkers, although the specific purpose of brief intervention may differ for each of these patient groups. Thus, three types of clinical situations exist in which brief intervention is used. First, brief intervention can help reduce alcohol use and the risk of alcohol-related problems in nondependent drinkers who consume alcohol amounts above the recommended limits (i.e., at-risk and problem drinkers). The goal of brief intervention with this population is to reduce alcohol use to low-risk levels, thereby minimizing the drinkers’ risk of developing alcohol-related social and medical problems. Thus, the aim of brief intervention for these drinkers is not so much abstinence as harm reduction. For example, in young men, total mortality and the relative risk^3^ of dying from violent causes increase with increasing alcohol consumption (see figure 1). Therefore, if brief intervention can reduce alcohol use in young men from five standard drinks (i.e., 60 grams of pure alcohol) per day to two standard drinks (i.e., 28 grams of pure alcohol) per day, those men’s relative risk for dying decreases fivefold (Andreasson et al. 1988). Similarly, the relative risk of developing and dying from liver cirrhosis rises with increasing alcohol consumption (figure 2) (Anderson et al. 1993) and could be reduced by brief intervention–associated reductions in drinking levels.

A second clinical situation in which brief intervention may be useful is to facilitate medication compliance and abstinence in patients who are being treated with pharmacological therapies for alcohol dependence and coexisting psychiatric conditions, such as depression. Failure to continue taking their medication (i.e. noncompliance) is a major issue with these patients, a problem that brief intervention may ameliorate. For example, O’Connor and colleagues (1997) found that alcohol-dependent patients treated with either disulfiram or naltrexone^4^ were more likely to respond to and remain on their medication if they also received brief counseling. Similar benefits of brief intervention were noted in people treated with antidepressant medications. As more effective pharmacological agents become available, brief intervention is likely to become an increasingly important treatment strategy.

The third clinical situation in which brief intervention is beneficial involves alcohol-dependent patients or patients with alcohol-related problems who do not respond to this intervention alone. Although brief intervention by itself is not sufficient in these cases, it can help health care providers identify those patients and refer them to specialized treatment. Most patients who are referred for an assessment of their alcohol-related problems or for counseling either do not schedule an appointment or fail to keep the scheduled assessment (Bernstein et al. 1997). In the terms of problem-behavior modification (Prochaska and DiClemente 1992), the patients are still at the “precontemplation stage” and have not yet reached the “action stage.” For patients in the precontemplation stage, the health care provider must address their ambivalence, resistance, and fears to ensure a successful referral. Brief intervention can greatly facilitate this process and increase the rates of successful assessment completion and admission to a treatment program (Bernstein et al. 1997).

## Relationship of Brief Intervention and Other Approaches

A variety of alcoholism treatment approaches exist, ranging from physician-delivered brief intervention to intensive inpatient therapy. Brief intervention is primarily based on motivational enhancement therapy. In addition, brief intervention uses many elements of 12-step-based methods and cognitive behavioral therapy. Nevertheless, brief intervention differs from these three types of interventions—which primarily are administered as long-term therapy—in two respects. First, brief intervention frequently is not aimed at achieving complete abstinence but focuses on harm reduction and on increasing the patient’s readiness to change his or her behavior. Second, the total number of visits during which brief intervention is delivered is limited.

Several strategies can help clinicians achieve their goals in the limited time-frame available for brief intervention:

- The physician can emphasize specific medical problems related to a patient’s alcohol consumption to raise the patient’s awareness that alcohol use can lead to serious health problems.
- The clinician can provide the patient with a written contract (in the form of a prescription) that specifies goals for reducing drinking levels.
- In many cases, if the physician has had a long-term and trusting relationship with the patient, the physician can influence the patient to change his or her behavior.

## Implementing Brief Intervention in Primary Care

Many studies have demonstrated that brief intervention delivered in a primary care setting can be an effective way to help at-risk or problem drinkers change their drinking behavior, thereby ameliorating or preventing alcohol-related health and other problems. Nevertheless, brief intervention has not yet been widely implemented in primary care settings. Several factors contribute to this lack of implementation.

First, health care settings today are complex systems involving numerous parties, including patients, health care providers, purchasers (e.g., employers and governmental agencies), and payers (e.g., insurance companies and health maintenance organizations). All of these parties have specific and sometimes competing agendas. For example, health care providers are primarily interested in providing the most effective care to their patients (including screening for potential health problems, such as excessive alcohol use), whereas purchasers and payers also are interested in cost containment. To enhance the implementation of brief intervention, health care purchasers and payers need to provide financial support and leadership. To achieve this goal, both purchasers and providers of health care insurance must realize that the prevention and treatment of alcohol problems will improve their clients’ health, thereby reducing both health care and social costs. Professional organizations must more actively work with payers and providers to allocate resources that accurately reflect the adverse effects of alcohol problems on the health care industry and on the health of the American people.

Second, many clinicians do not receive adequate skills training in conducting brief intervention. Furthermore, clinicians often are not compensated or rewarded for conducting clinical activities related to the prevention and treatment of alcohol problems. Several steps can help remedy those barriers. For example, clinicians should attend training workshops on how to make brief intervention for alcohol problems an essential component of their regular clinical activities. The workshops should focus on skills-training activities, using role-play exercises and standardized patients (i.e., lay people trained to consistently replicate a clinical encounter). Quality improvement programs, which are being implemented throughout the health care system, also can provide a unique opportunity to change clinician practice behavior. Many of these programs currently do not cover alcoholism screening and intervention; however, the establishment of monitoring systems to examine alcohol use in patients being treated for hypertension, depression, or anxiety disorders could significantly change practice patterns. Such incentives as financial reimbursement, paid education time to attend training workshops, and quality-improvement peer review programs also may encourage clinicians to implement alcohol screening and intervention in their practices.

Third, brief intervention may be particularly difficult to implement in clinic settings, which already must accommodate a wide range of clinical tasks and activities, such as routine physicals, treatment of acute medical problems (e.g., trauma, infections, anxiety, and headaches), management of chronic conditions (e.g., depression, hypertension, and diabetes), and prevention programs (e.g., breast cancer screening, nutrition and diet counseling, and immunizations). To implement and maintain alcohol screening and intervention in clinic settings, procedures must be developed to incorporate brief intervention into routine clinical care. Measures such as self-administered screening tests, incorporation of alcohol-related questions in the assessment of routine vital signs, and computerized reminder systems alerting clinicians to screen clients for alcohol problems can help identify patients who may benefit from brief intervention. Additional reminders (e.g., for followup) can be attached to the clients’ medical records or posted in another prominent location. Self-help booklets, alcohol consumption diary cards, lists of self-help group meetings (e.g., Alcoholics Anonymous), and referral information with telephone numbers and names of alcoholism treatment specialists can assist clinicians and clients in establishing followup plans and strategies.

Fourth, the implementation of brief intervention often is prevented by the lack of integration of alcohol and other drug (AOD) treatment into primary care systems. Alcoholism treatment has historically occurred outside the traditional medical care system, and many alcoholism treatment programs are free standing and community based. Lack of communication between such specialized treatment programs and the client’s primary health care providers can have serious adverse effects on a patient’s long-term sobriety. For example, in contrast to other specialty referral systems (e.g., medical and surgical specialty clinics), AOD treatment programs do not routinely send copies of the assessment, treatment plan, or discharge summary to the client’s health care provider. Similarly, AOD treatment specialists do not routinely call the client’s physician or therapist to coordinate and jointly develop long-term treatment plans. Concerns about patient confidentiality may prevent the free flow of information between the specialized treatment program and the primary health care provider. Clients with alcohol problems, however, deserve treatment providers who communicate and work together to provide coordinated, comprehensive care.

Consequently, the integration of specialized alcoholism treatment into the general medical care system is an important component in enhancing the implementation of brief intervention. Two measures can facilitate an integrated treatment process of brief intervention and referral to specialized treatment:

- Alcoholism treatment programs should be located in close physical proximity to primary care offices, because physicians are more likely to refer clients to and communicate with a trusted colleague whose office is down the hall rather than make referrals to a stranger whose practice is located many miles away in a different system of care. Local proximity between primary care and specialized care providers also makes it easier for clients to accept and follow through with a referral.
- Communication between primary care and alcoholism treatment providers can be increased by encouraging primary health care providers to send referral letters to and request clinical updates from alcoholism treatment programs. Conversely, treatment programs should be encouraged to have their patients sign medical release forms that allow the programs to send assessments and treatment plans back to the primary health care provider. Such releases also allow primary care providers and alcoholism counselors to communicate directly by telephone to discuss treatment options. Ideally, primary care physicians and AOD treatment specialists should be members of one comprehensive medical care team.

## Summary

Brief interventions are counseling strategies that primary care physicians can deliver during routine office visits to help clients change their drinking behavior (or any other health-related behavior). Numerous studies have suggested that brief intervention can reduce alcohol consumption in a substantial number of at-risk or problem drinkers and can facilitate the referral of dependent drinkers into specialized alcoholism treatment. As a result, brief intervention can help prevent or ameliorate numerous alcohol-related medical and social problems and the associated costs. Despite the encouraging results regarding the effectiveness of brief intervention, however, such measures have not yet been widely incorporated into primary care practices. Factors contributing to this lack of implementation include the complexity of the health care system, inadequate physician training, and lack of integration of AOD treatment into general medical practice. Coordinated efforts of health care providers, purchasers, and payers can help remove these barriers, thereby ensuring that patients with alcohol-related problems receive the comprehensive care they need.

## Footnotes

1 The CAGE, a widely used screening test for alcoholism, includes the following four questions: (1) Have you ever felt you should Cut down on your drinking?; (2) Have people Annoyed you by criticizing your drinking?; (3) Have you ever felt Guilty about your drinking?; and (4) Have you ever had a drink first thing in the morning to steady your nerves or to get rid of a hangover (Eye opener)?

2 Sample copies of intervention workbooks and drinking diary cards, which were developed for Project TrEAT (Trial for Early Alcohol Treatment), are available from the authors of this article.

3 The “relative risk” is the ratio of the incidence of an event (e.g., a violent death) in alcohol-exposed people to the incidence of that event in nonexposed people.

4 Disulfiram is a medication that causes an unpleasant reaction (e.g., flushing, nausea, and vomiting) when a person ingests alcohol. Naltrexone can reduce craving for alcohol in alcohol-dependent people.
